# Conditions for the Joint Conversion of CO_2_ and Syngas in the Direct Synthesis of Light Olefins Using In_2_O_3_–ZrO_2_/SAPO-34 Catalyst

**DOI:** 10.1021/acs.iecr.1c03556

**Published:** 2021-11-09

**Authors:** Ander Portillo, Ainara Ateka, Javier Ereña, Andres T. Aguayo, Javier Bilbao

**Affiliations:** Department of Chemical Engineering, University of the Basque Country UPV/EHU, P.O. Box 644, Bilbao 48080, Spain

## Abstract

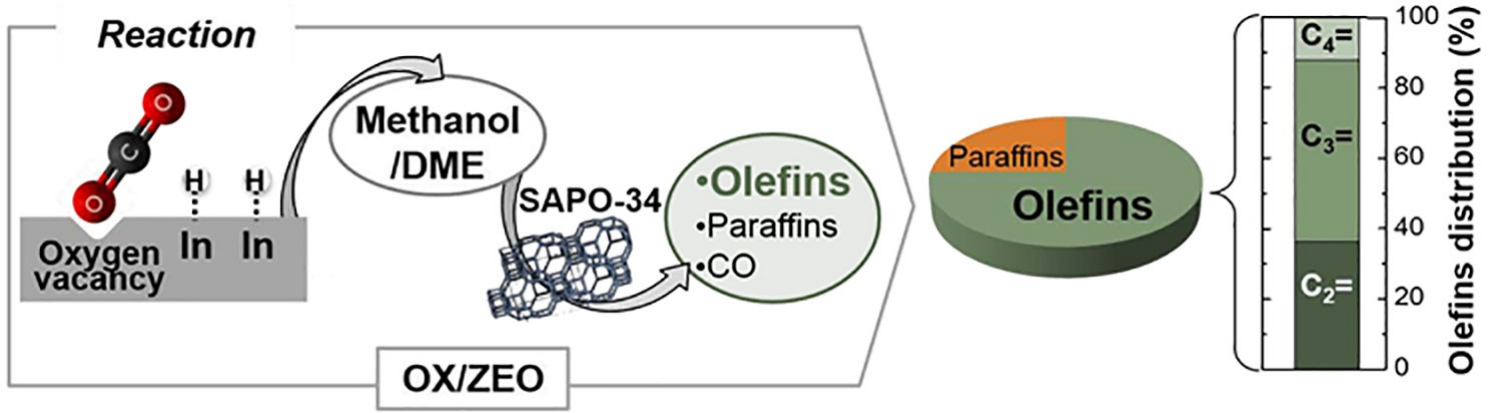

The conditions for promoting the joint conversion of CO_2_ and syngas in the direct synthesis of light olefins have been studied. In addition, given the relevance for the viability of the process, the stability of the In_2_O_3_–ZrO_2_/SAPO-34 (InZr/S34) catalyst has also been pursued. The CO+CO_2_ (CO_*x*_) hydrogenation experimental runs were conducted in a packed bed isothermal reactor under the following conditions: 375–425 °C; 20–40 bar; space time, 1.25–20 g_catalyst_ h mol_C_^–1^; H_2_/(CO_*x*_) ratio in the feed, 1–3; CO_2_/(CO_*x*_) ratio in the feed, 0.5; time on stream (TOS), up to 24 h. Analyzing the reaction indices (CO_2_ and CO_*x*_ conversions, yield and selectivity of olefins and paraffins, and stability), the following have been established as suitable conditions: 400 °C, 30 bar, 5–10 g_cat_ h mol_C_^–1^, CO_2_/CO_*x*_ = 0.5, and H_2_/CO_*x*_ = 3. Under these conditions, the catalyst is stable (after an initial period of deactivation by coke), and olefin yield and selectivity surpass 4 and 70%, respectively, with light paraffins as byproducts. Produced olefin yields follow propylene > ethylene > butenes. The conditions of the process (low pressure and low H_2_/CO_*x*_ ratio) may facilitate the integration of sustainable H_2_ production with PEM electrolyzers and the covalorization of CO_2_ and syngas obtained from biomass.

## 1 Introduction

It is well established that replacing fossil sources for renewable energies is the solution to reverse the climate change caused by greenhouse gas emissions (in particular by CO_2_).^1^ However, changing the energy model requires a transition period, and the duration of this is conditioned by economic factors and by the increase in energy demand related to social development.^2^ In this scenario, it is necessary to control the fluxes of carbon between the different geo-habitats^3^ and to activate carbon capture and utilization (CCU) strategies for a long-term sustainable world.

The technological development of efficient routes for the large-scale conversion of CO_2_ into value-added products is imperative (to offset the cost of its capture and storage) to facilitate the viability of CCU strategies. This requires activating the stable structure of CO_2_ generating C–C, C–H, C–O, and C–N bonds.^4^ In addition, biomass gasification and pyrolysis derivatives (syngas and bio-oil, respectively) offer good prospects to replace fossil sources, helping to reduce CO_2_ emissions. Hansen et al.^5^ and Kargbo et al.^6^ have made reviews of the state of the art of these technologies and a comparison of their techno-economic feasibility and sustainability, respectively. Among the routes for valorizing syngas and CO_2_, whether joint or separately,^7^ the best prospects for short-term scaling correspond to the catalytic processes,^8^ particularly those of hydrogenation at high pressure for the production of methanol,^9^ liquid fuels, and raw materials for the petrochemical industry (mainly olefins and aromatics).^10^ It should be noted that the strategies of the catalytic processes developed for the conversion of syngas or CO_2_ into hydrocarbons are similar, and indirect and direct routes can be distinguished. The indirect route requires two reaction stages: first, synthesis of methanol/DME, and subsequent transformation into hydrocarbons in a second reactor. The development of catalysts for methanol synthesis from CO_2_ is outstanding,^11^ with those based on Cu/ZnO/Al_2_O_3_ being the most used alternative.^12^ The synthesis of methanol is an ideal process to be integrated with CO_2_ capture in conventional cement plants,^13^ and methanol is converted into light olefins through the MTO (methanol-to-olefin) process.^14^ For this, a fluidized bed reactor with catalyst (SAPO-34) circulation is used.^15^ However, the synthesis of DME offers thermodynamic advantages (by integrating the synthesis of methanol and its dehydration in the same reactor), and consequently, CO_2_ conversion is higher than in the synthesis of methanol.^16^ In addition, the cofeeding of syngas derived from biomass gasification is more feasible.^17^ A bunch of bifunctional catalysts have been developed for DME synthesis,^18^ which can be later converted into hydrocarbons on an HZSM-5-based catalyst.^19^ This catalyst can be designed for the selective production of olefins or gasoline, and can be reused in reaction–regeneration cycles.^20^

In the direct route, in one stage, the production of hydrocarbons from CO_2_ is conducted in a single reactor.^21^ With the proper selection of a bifunctional catalyst and reaction conditions, the direct and selective synthesis of olefins can be achieved through two alternative routes:^22^ (i) Fischer–Tropsch synthesis (FTS) (Anderson–Schulz–Flory mechanism).^23^ The incorporation of an acid catalyst together with the metallic catalyst composed of Fe or Co for the in situ conversion of the mixture of synthesized hydrocarbons into olefins,^24^ and; (ii) with methanol/DME as intermediates, with OX/ZEO (metal oxide/zeolite) catalysts, whose metallic function catalyzes the reactions of methanol/DME synthesis and the acid function the in situ conversion of these oxygenates into olefins. The route has been proposed for syngas conversion^25^ and afterward for CO_2_.^26^ A simplified reaction scheme of this route is shown in eq 1:
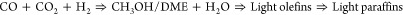
1The implementation of the second stage in the scheme in eq 1 (conversion of methanol/DME into hydrocarbons) in the same reactor used for oxygenate synthesis is interesting not only for reducing the capital cost of the two-stage process but for displacing the thermodynamic equilibrium of methanol/DME synthesis, favoring the conversion of CO_2_ and CO. It should be noted that the economic viability of the catalytic processes for CO_2_ hydrogenation is conditioned by the economic profitability and feasibility of the sustainable generation and storage of H_2_.^27^ In this regard, the lower pressure required in the direct process facilitates integrating the reaction with commercial PEM electrolyzers, which supply hydrogen at 15–30 bar.^28^ However, the synergies derived from integrating the stages of methanol/DME synthesis and its conversion into hydrocarbons and the fact that the reaction must be carried out under intermediate conditions of those ideal for each of the individual stages, hampers understanding the reaction mechanism. Nonetheless, a mechanism with formate ions from CO_2_ and formyl ions from CO as intermediates,^29^ as the role of H_2_O is relevant in the medium, is reasonably justified in the synthesis of methanol/DME. Likewise, the dual cycle mechanism for the conversion of these oxygenates into hydrocarbons is reasonably justified.^30^ In particular, this second stage requires temperatures above 375 °C,^16^ and for such high temperatures, methanol/DME synthesis is hampered.^31^ Consequently, the progress of methanol/DME conversion has a key role to achieve remarkable conversion of CO and CO_2_. However, there are other factors that hinder the understanding of the results expected from the scheme in eq 1, which simplifies the reality of a complex reaction system. Among others: (i) the evolution of the water gas shift (WGS) reaction, which relates the concentration of the key components in the reaction scheme (CO, CO_2_, H_2_, H_2_O); (ii) the different reactivity of CO and CO_2_;^32^ (iii) the different reactivity of methanol and DME;^33^ (iv) the complex role of H_2_O formed in the oxygenate conversion, displacing the WGS reaction and attenuating oxygenates synthesis reactions^34^ and their conversion into olefins, but also attenuating the deactivation of the catalyst by coke.^35^ As a result, it is difficult to predict the effect of these features on the results, and thus the suitable operating conditions must be experimentally determined along with the selection of the catalyst.

The performance (activity, selectivity, and stability) of the bifunctional OX/ZEO catalyst will be determined by the composition and properties of its components. Thus, the presence of oxygen vacancies in the metallic function is a key feature for the adsorption of CO and CO_2_.^36^ In addition, in the dual cycle mechanism, hydrocarbon distribution depends on the acidity and shape selectivity of the acid function,^37^ and consequently, the acid strength of the sites, the zeolite cavity, and the pore size control the selectivity of olefins or gasoline.^38^ The ideal composition of the OX/ZEO bifunctional catalyst to maximize the selectivity of olefins in the hydrogenation of CO_2_ and CO+CO_2_ mixtures also requires avoiding the ability of the metallic function to overhydrogenate the double C=C bonds, which forms methane.^39^ On this basis, In_2_O_3_–ZrO_2_/SAPO-34 catalyst shows good prospects for the selective production of olefins from CO_2_ in a remarkable reaction rate.^40^ The high methanol synthesis activity of In_2_O_3_ is a consequence of its CO_2_ adsorption capacity in the superficial oxygen vacancies.^41^ This activity and the high olefin selectivity have also been related to the suppression of the formation of CO as a byproduct^42^ and to the limited capability of In_2_O_3_ to overhydrogenate the C =C bonds and to form methane.^43^ The CO_2_ adsorption, competing with H_2_, is conditioned by the location and stability of the oxygen vacancies,^44^ directing the process toward methanol formation (linear adsorption) or CO formation (bent adsorption) through the reverse water gas shift (rWGS) reaction.^45^ Wang et al.^46^ developed a mechanism in which the structural evolution of In_2_O_3_ was determined to be the key feature. This evolution has been determined by in situ monitoring of the catalyst in operation.^47^ As to ZrO_2_, it has various roles in the metallic phase. On the one hand, it acts as a structural promoter to attenuate the sintering of In_2_O_3_, and on the other, it also leads to maintaining oxygen vacancies on the surface through electronic interactions at the interface that help CO_2_ adsorption^41^ and further accelerate methanol production.^48^

As to the acid function of the catalyst, it is well established that SAPO-34 (CHA topology, in which spacious cavities (10 × 6.7 Å) are connected by small (3.8 × 3.8 Å) 8-ring cages)^49^ is suitable for selectively producing light olefins from methanol/DME. This reaction is industrially carried out at atmospheric pressure and without H_2_ in the medium, and under these conditions, the deactivation of SAPO-34 is very fast.^50^ The blockage of the cavities of SAPO-34 by coke, limiting the diffusion of the products, is the cause for deactivation.^51^ This phenomenon requires a limited residence time of the catalyst in the reactor and its regeneration in a separate unit.^52^ However, the coke deactivation of SAPO-34 has been reported to be limited above 360 °C on syngas conversion into hydrocarbons, because of the effect of the high partial pressure of H_2_ for attenuating coke formation.^53^

Tan et al.^42^ studied the effect of the addition of CO as promoter given its interest from the perspective of recycling in an industrial process of CO_2_ hydrogenation. These authors verified that the addition led to increases in the conversion of CO_2_ and the yield and selectivity of olefins, which is explained by the fact that the presence of CO affects the thermodynamic equilibrium, attenuating the extent of the rWGS. In the present work, the joint conversion of CO_2_ and syngas has been assessed in a wide range of conditions, aiming to determine the synergistic effect of the cofeeding and the appropriate conditions for the selective and stable production of light olefins. The interest in the cofeeding is based on the fact that two strategies for reducing CO_2_ emissions are combined, as syngas can be obtained via gasification of biomass or wastes (plastics, tires). Furthermore, with the cofeeding, the H_2_ requirement corresponding to the hydrogenation of CO_2_ (key feature for the viability of the process) is partially provided by the syngas. Consequently, the joint conversion of CO_2_ and syngas into hydrocarbons may be more interesting from an environmental point of view than the individual conversion of the two streams. With these objectives, the performance of In_2_O_3_–ZrO_2_/SAPO-34 (InZr/S34) catalyst has been studied for different operating conditions (temperature, pressure, space time, H_2_/CO_*x*_ molar ratio in the feed), paying attention to CO_2_ and CO+CO_2_ mixture (CO_*x*_) conversions, hydrocarbon fraction (light olefins and paraffins) yields and selectivities, and their evolution with time on stream.

## 2 Experimental Section

### 2.1 Catalyst Preparation

In_2_O_3_–ZrO_2_ (InZr) function was synthesized following a conventional coprecipitation method, based on that reported in previous works for the preparation of other metallic functions with conventional^54^ and core–shell^55^ structure. A metal nitrate solution of (In(NO_3_)_3_ (Sigma-Aldrich) and Zr(NO_3_)_4_ (Panreac) with the desired In/Zr ratio of 2 (1 M) was coprecipitated under stirring with ammonium carbonate (Panreac, 1 M) at 70 °C and neutral pH. The mixture was aged for 2 h to ensure the complete precipitation, filtered and cleaned several times with deionized water, and subsequently dried and calcined at 500 °C (selected on the basis of the literature values^39^) for 1 h. In a recent work, Numpilai et al.^56^ ascertained the relevance of calcination temperature on the preparation of In_2_O_3_–ZrO_2_ catalysts for the synthesis of methanol from CO_2_. These authors reported the maximum methanol yield for calcination temperatures between 800 and 900 °C for reactions carried out within the 320–340 °C. Finally, the resulting powder was pelletized, crushed, and sieved to the desired particle size (125–250 μm). The final InZr/S34 bifunctional catalyst was obtained by physical mixture of the previously detailed In_2_O_3_–ZrO_2_ function and a commercial SAPO-34 acid function (ACS Material, pelletized into 300–400 μm particles) with a In_2_O_3_–ZrO_2_/SAPO-34 mass ratio of 2/1. This configuration was determined to be optimal among other options (individual beds in series, a bifunctional catalyst prepared by pelletizing mortar-mixed In_2_O_3_–ZrO_2_+SAPO-34 powder), coinciding with that reported by other authors.^39,57^

### 2.2 Catalyst Characterization

The textural properties of the metallic and acid functions of the catalyst (Table 1) have been determined by N_2_ adsorption–desorption analyses (Micromeritics ASAP 2010) at −196 °C. The acidity has been determined by temperature-programmed desorption (TPD) of NH_3_ (Micromeritics Autochem 2920). The results confirm the highest porosity and acidity of SAPO-34 compared to the bulk oxide.

**Table 1:** Physical and Acid Properties of the Metallic and Acid Functions of the Catalyst

	textural properties				
catalyst	*S*_BET_(m^2^ g^–1^)	*V*_micropore_ (cm^3^ g^–1^)	*V*_pore_ (cm^3^ g^–1^)	dp (nm)	total acidity (mmol_NH3_ g_cat_^–1^)
InZr	58.2	0.0027	0.23	9.0	122.6
SAPO-34	651.8	0.2192	0.23	1.5	777.6

**Figure 1:**
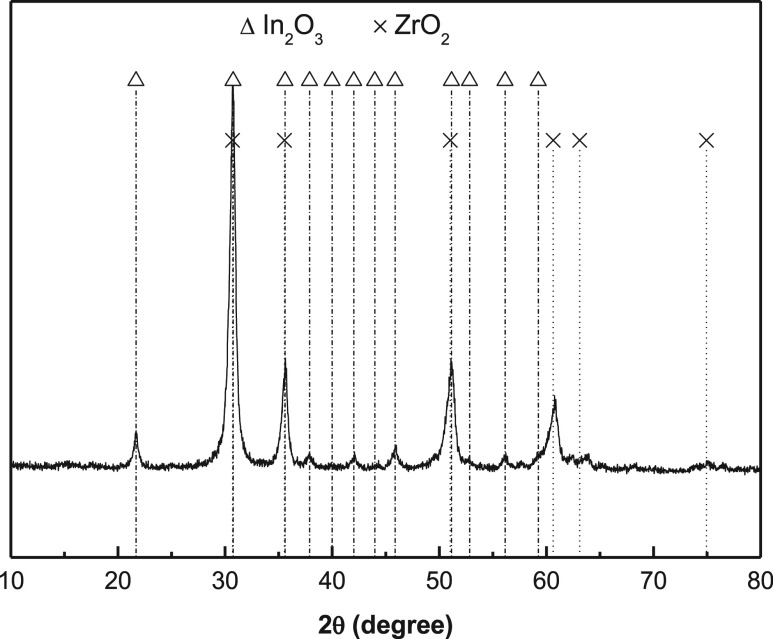
In_2_O_3_–ZrO_2_ function XRD.

Regarding the chemical composition and structure of the catalyst, X-ray diffraction (PANalytical Xpert PRO) and X-ray fluorescence (PANalytical Axios) analyses have been carried out. The XRD patterns plotted in Figure 1 reveal the presence of both In_2_O_3_ and ZrO_2_ structures. The proximity of the peaks, resulting from the same crystalline structure of both phases (cubic/bixbyite), hampers the clear detection of the migration of one metal to the structure of the other from the XRD pattern. As such, Rietveld analyses were conducted to reveal the interaction between the metals. That is, the presence of In was observed in the structure of ZrO_2_ (in a lesser extent) and Zr was observed in the structure of In_2_O_3_. Moreover, Numpilai et al.^56^ proved using H_2_-TPD that the interaction between In and Zr metals modified the electronic properties of In_2_O_3_/ZrO_2_ catalysts compared to those of In_2_O_3_ and ZrO_2_. As a result, besides the oxygen vacancies the In_2_O_3_ structure has itself, the replacement of some In atoms by Zr atoms gives way to the formation of additional oxygen vacancies because of the different valence number of both.^58^ XRF results confirm the presence of Zr in a In/Zr molar ratio of 2.03.

The coke deposited on the catalyst during the reactions has been analyzed by means of temperature programmed oxidation (TPO) in an air atmosphere (20 cm^3^ min^–1^) up to 700 °C at a heating rate of 7 °C min^–1^ in a TGA Q5000 IR thermobalance (TA Instruments). The content of solid material deposited on the used catalysts has been determined by integrating the area under the TPO profiles.

### 2.3 Reaction and Analysis Equipment

The catalytic runs have been carried out in automated reaction equipment (PID Eng & Tech Microactivity Reference) provided with an isothermal fixed bed reactor. The reactor is made of 316 stainless steel (with an internal diameter of 9 mm and an effective length of 10 cm) and coated with a ceramic layer to avoid the direct contact of the reaction components with the steel and avoid any possible side reaction. This equipment enables working at pressures up to 100 bar and temperatures up to 700 °C. In the catalytic bed, the catalyst is diluted in SiC (0.035 mm particle size), an inert solid, to ascertain the isothermal condition of the bed and to attain a suitable bed height when operating at small space time values.

The feedstock and product streams were analyzed in a micro chromatograph (Varian CP-4900, Agilent) that was equipped with three analysis modules composed of TCD detectors and different chromatographic columns: (i) Porapak Q (PPQ) (10 m × 20 μm) for the quantification of CO_2_, methane, H_2_O, C_2_–C_4_ hydrocarbons, methanol, and dimethyl ether; (ii) molecular sieve (MS-5) (10 m × 12 μm) for the quantification of H_2_, N_2_, O_2_, and CO; (iii) 5 CB column (CPSiL) (8 m × 2 μm) for the quantification of C_4+_ hydrocarbons.

The reaction runs have been carried out under a wide range of operating conditions: 375–425 °C; 20–40 bar; space time, 1.25–20 g_catalyst_ h mol_c_^–1^; H_2_/(CO+CO_2_) ratio in the feed, 1–3; CO_2_/(CO+CO_2_) ratio in the feed, 0.5; time on stream (TOS), up to 24 h. Table 2 lists the individual catalyst mass loadings corresponding to each space time value used in the experimental runs.

**Table 2:** Catalyst Loading for Different Space Time Values Used

space time (g_cat_ h mol_C_^–1^)	In_2_O_3_–ZrO_2_/SAPO-34 (mg)	In_2_O_3_–ZrO_2_ (mg)	SAPO-34 (mg)
1.25	45.9	30.6	15.3
5	188.9	122.6	61.3
10	367.8	245.2	122.6
20	735.6	490.4	245.2

### 2.4 Reaction Indices

The conversion of the CO+CO_2_ mixture, denoted as CO_*x*_, has been defined as
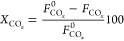
2where *F*_CO_*x*__^0^ and *F*_CO_*x*__ are the molar flow rates of the CO_*x*_ at the inlet and outlet of the reactor, respectively.

CO_2_ conversion has been defined analogously:
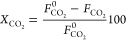
3where *F*_CO_2__^0^ and *F*_CO_2__ are the CO_2_ molar flow rates at the inlet and outlet of the reactor, respectively.

Yield and selectivity (*Y*_*i*_ and *S*_*i*_, respectively) of every carbonated product (excluding CO and CO_2_), that is, C_2_–C_4_ olefins, C_2_–C_4_ paraffins, methane, and oxygenates (methanol and DME), have been defined as
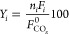
4
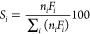
5where *n*_*i*_ refers to the number of C atoms in a molecule of component *i* and *F*_*i*_ to the molar flow rate of component *i* at the reactor outlet stream.

## 3 Results

In this section, the effect of reaction temperature, pressure, space time, and feed H_2_/CO_*x*_ molar ratio on the reaction indices is studied. The influence of these operating variables on the conversion of CO_2_ and of the CO+CO_2_ mixture and on product distribution and their evolution with time on stream will be assessed to determine the most suitable operating conditions for catalyst stability and maximizing olefin production.

### 3.1 Temperature

#### 3.1.1 CO_*x*_ Conversion, Yields, and Selectivities

**Figure 2:**
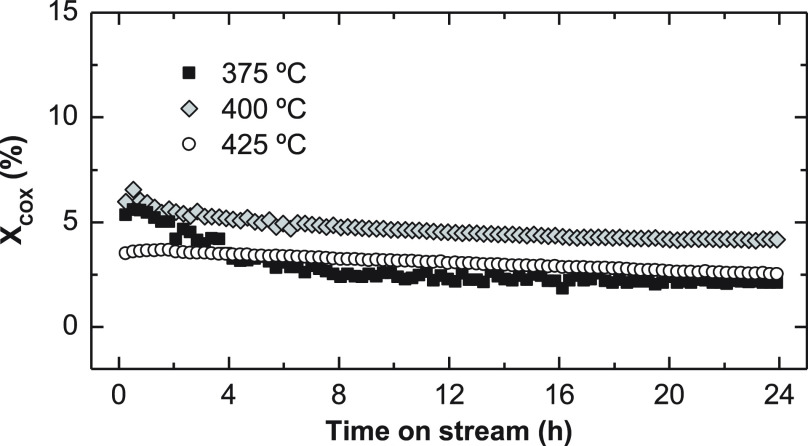
Effect of temperature on CO_*x*_ conversion. Reaction conditions: 30 bar; 5 g_cat_ h mol_C_^–1^; CO_2_/CO_*x*_, 0.5; H_2_/CO_*x*_, 3; TOS, 24 h.

**Figure 3:**
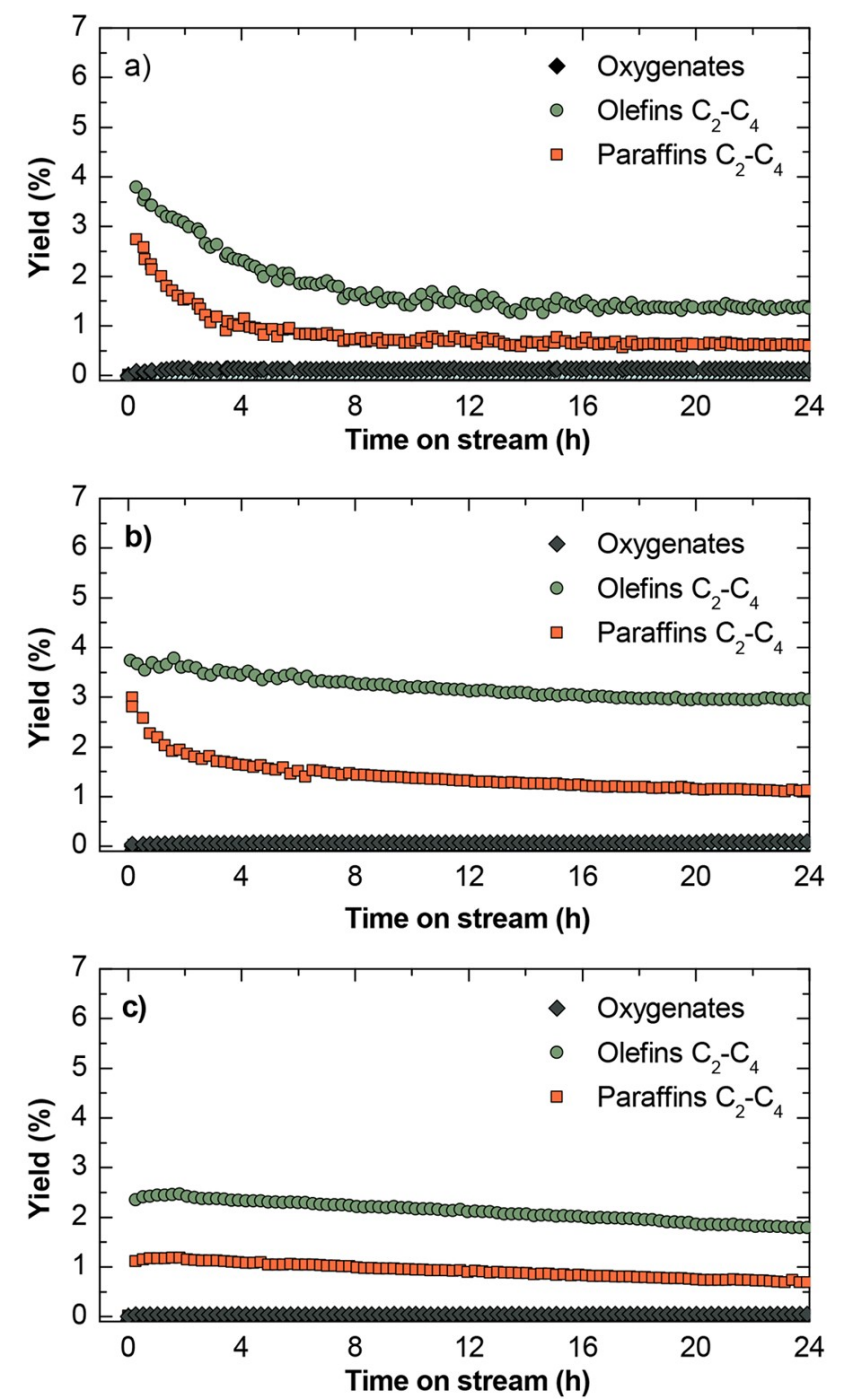
Evolution of the yield of products with time on stream at (a) 375, (b) 400, and (c) 425 °C. Reaction conditions: 30 bar; 5 g_cat_ h mol_C_^–1^; CO_2_/CO_*x*_, 0.5; H_2_/CO_*x*_, 3; TOS, 24 h.

The evolution of CO_*x*_ conversion with time on stream is plotted in Figure 2 for three studied temperatures. Even if the results shown correspond to specific operating values, the trends are qualitatively similar for all other cases. The results corresponding to product yields are shown in Figure 3 (and selectivities in Figure S1).

The results in Figures 2 and 3 show the relevance of reaction temperature due to the effect of this variable on the extent of each reaction stage and on catalyst deactivation. Methanol/DME conversion (the second reaction stage) is favored with increasing temperature within the studied range. Furthermore, the presence of high H_2_O quantities (up to 4%) on the reaction medium (generated via rWGS and via methanol/DME dehydration) diminishes the activity of the catalyst,^35^ requiring a higher temperature. However, higher temperature has an opposite effect in two reactions influencing CO_*x*_ conversion, as observed in the hydrogenation of CO_2_ to methanol/DME.^41^ On the one hand, according to thermodynamics, it hinders the extent of methanol synthesis^31^ and DME synthesis,^16^ which are key reactions in the first stage, but on the other hand, it favors the rWGS reaction according to thermodynamics.^59^ As a consequence of these effects, at zero time on stream, CO_*x*_ conversion reaches its maximum value at 375–400 °C, being slightly higher at 400 °C (Figure 2). Note that the data correspond to time on stream values starting from 20 min. Previously, an initiation period was observed with apparent increasing activity of the catalyst. This period is characteristic of both reaction stages. On the one hand, for methanol/DME synthesis over an In_2_O_3_ catalyst, this period has been reported to be related to the formation of the active oxygen vacancies.^47^ These vacancies are generated by removing surface oxygen atoms and reducing In_2_O_3_ to In_2_O_3–*x*_ (leading to different energetic barriers depending on the vacancy location) in a H_2_ or CO atmosphere (or through thermal treatment). Bielz et al.^60^ determined that oxygen vacancies formed either from H_2_ or CO can only be reduced to a small extent by CO_2_ or H_2_O,^41^ giving In_2_O_3_ a unique redox property. During methanol formation, a cyclic creation and annihilation of oxygen vacancies takes place, as determined by various authors experimentally for In_2_O_3_^41^ and In_2_O_3_–ZrO_2_^61^ catalysts. Using periodic density functional theory (DFT) calculations, Ye et al.^62^ examined six possible surface oxygen vacancies and determined that CO_2_ hydrogenation to formate (HCOO*) is more favorable than protonation to bicarbonate species.^46^ Overall, two pathways stand out to explain the mechanism on the bifunctional catalyst.^63^ CO_2_ would adsorb placing one of its oxygen atoms in a vacancy of the metallic surface and would be hydrogenated by In–H to form HCOO* species after H_2_ being dissociatively adsorbed. Then, HCOO* will react with H* to produce H_2_COO* species, which will be hydrogenated to H_3_CO* methoxy species, which will further hydrogenate to form methanol or DME.^64^ This mechanism was confirmed by Frei et al.^59^ On the other hand, over the acid function, methanol/DME is converted into hydrocarbons through the dual cycle mechanism, with a characteristic initiation period related to the time required for the formation of active intermediates in both cycles.^37^

The higher conversion at 400 °C (even though it is a moderate value, <7% in these reaction conditions) suggests that this is an adequate temperature to activate the oxygenate conversion pathway on the SAPO-34. In fact, oxygenate concentration on the product stream is negligible at 400 °C (0.071%, Figure 3b) and 425 °C (0.044%, Figure 3c), implying a complete conversion of methanol/DME. Moreover, this temperature is also suitable for the MTO process (methanol-to-olefin) on the SAPO-34 catalyst.^14^ It should be noted that this conversion value is not overtaken at higher space time values. These results reveal that methanol/DME synthesis is the limiting reaction stage in the scheme in eq 1, because of the aforementioned thermodynamic constraints for the CO and CO_2_ hydrogenation stage. In addition, at 400 °C, the olefin yield reaches almost 4% initially (Figure 3b). The undesired partial hydrogenation of the formed olefins yields light paraffins as byproducts, whereas methane has not been detected in any condition. Comparing the results with those obtained with the most alike catalysts available in the literature, the olefin yield is consistent with the results reported by Dang et al.^48^ (at 380 °C, 30 bar) and by Numpilai et al.^65^ (at 360 °C, 25 bar) with H_2_/CO_2_/N_2_ feedstocks. In other works,^48^ a higher space time is required to reach the same value of light olefin yield. Taking into account the different ratios between In_2_O_3_ and SAPO-34 functions, in our case, it requires a 2.5 times lower amount of In_2_O_3_ function and a 5 times lower amount of SAPO-34 function to attain a similar level of light olefin yield.

As expected, temperature also has a noticeable effect over catalyst deactivation. It is observed in Figure 3a, b (corresponding to 375 and 400 °C, respectively) that the deactivation rate decreases with increasing temperature. Indeed, the stability of the In_2_O_3_–ZrO_2_ function of the catalyst at such a high temperature as 400 °C should be pointed out. The stability is an important property of the catalyst taking into account that Cu-based catalysts (CuO-ZnO-Al_2_O_3_) usually used in the synthesis of methanol undergo a notable Cu sintering above 300 °C.^54^ It is also to be mentioned that according to the evolution of the results with time on stream depicted in Figures 2 and 3, there is evidence that the deactivation rate decreases progressively, trending the catalyst activity to a pseudosteady state, with a remarkable constant remaining activity. A pseudoequilibrium between coke precursor formation and elimination justifies this trend.^66^ This situation is interesting for scaling up the process, by prolonging catalyst lifetime prior to its regeneration.

**Figure 4:**
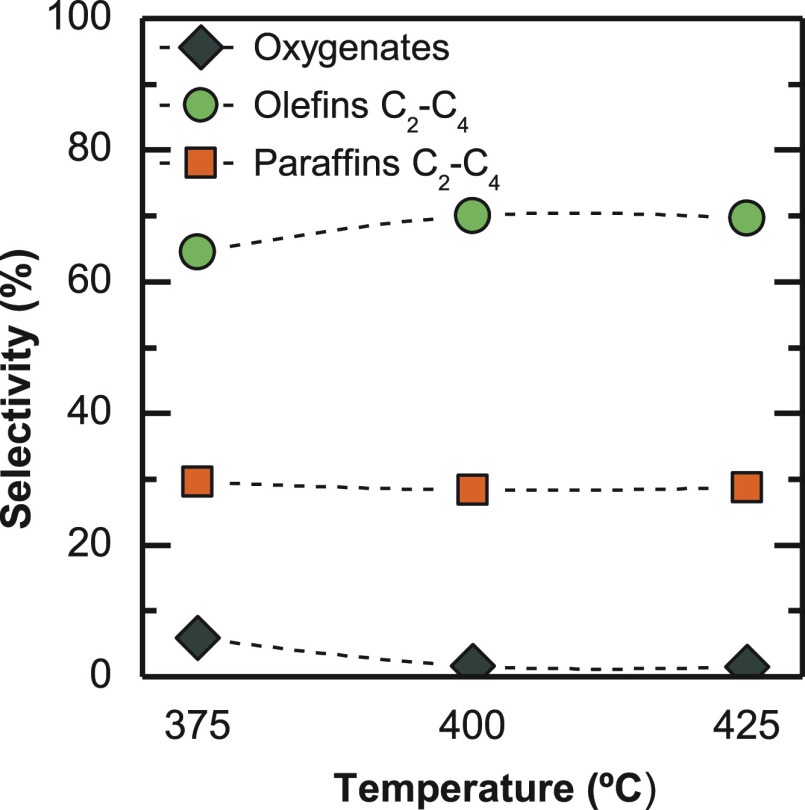
Temperature effect on product selectivity. Reaction conditions: 30 bar; 5 g_cat_ h mol_C_^–1^; CO_2_/CO_*x*_, 0.5; H_2_/CO_*x*_, 3; TOS, 16 h.

For the viability of the process on a larger scale, olefin selectivity is a key feature. Therefore, given the low per-pass conversion, the reactants must be recycled after separating the hydrocarbon products as to boost conversion, as in the methanol synthesis process.^67^Figure 4 shows the effect of temperature on the selectivity of light olefins and light paraffins (evolution with TOS on Figure S1). According to these data, a slight increase in the selectivity of light olefins is observed with increasing temperature from 375 to 400 °C. The results correspond to a pseudosteady state of the catalyst (TOS = 16 h). Furthermore, other hydrocarbons with more than four carbon atoms have not been detected on the product stream, as their formation is restricted by the small size of SAPO-34 catalyst cages.

**Figure 5:**
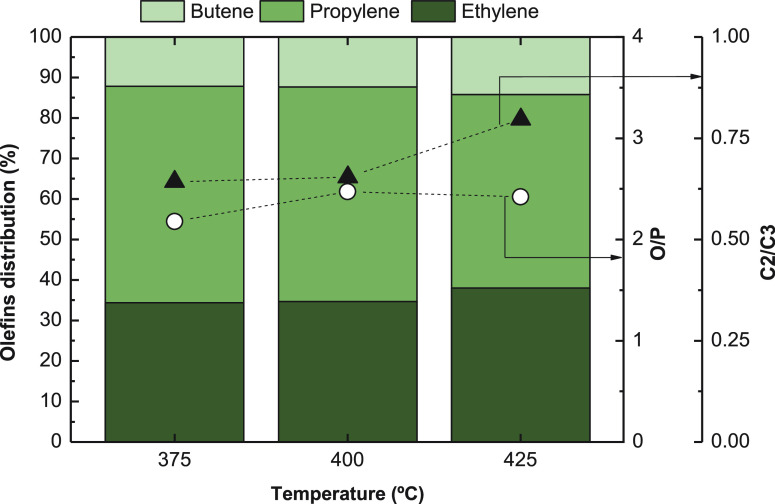
Effect of temperature on olefin distribution and on olefin/paraffin ratio Reaction conditions: 30 bar; 5 g_cat_ h mol_C_^–1^; CO_2_/CO_*x*_, 0.5; H_2_/CO_*x*_, 3; TOS, 16 h.

As for olefin distribution (Figure 5), it can be observed that propylene is the main product for all temperatures and butene percentage does not exceed 15% in any case. It is also observed, that the ethylene/propylene ratio slightly increases with temperature, analogously to the methanol/DME conversion to olefins over SAPO-34 catalysts.^68^ The higher propylene yield in these reaction conditions indicates the further advance of the alkene cycle with respect to that of aromatics,^69^ whereas the increase in ethylene selectivity at 425 °C is consistent with the results well established in the literature for methanol^70^ and DME^71^ conversion into olefins. Olefin distribution in these reactions is the consequence of the extent of the oligomerization-cracking mechanism, which leads to ethylene being the final olefin at high temperature.

#### 3.1.2 CO_2_ Conversion

**Figure 6:**
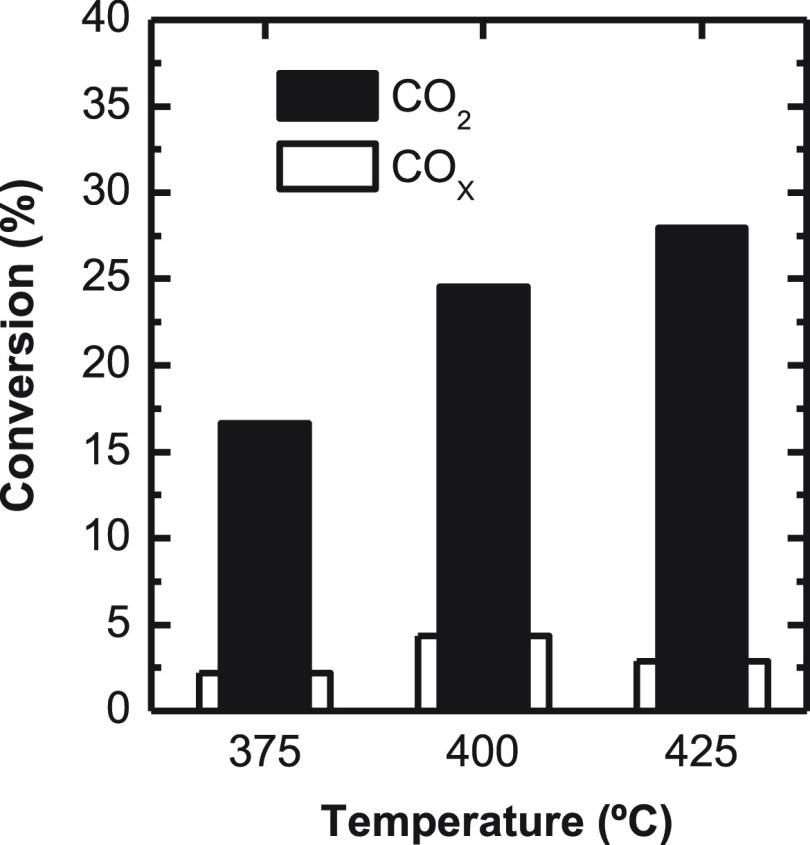
Temperature effect on CO_2_ and CO_*x*_ conversion. Reaction conditions: 30 bar; 5 g_cat_ h mol_C_^–1^; CO_2_/CO_*x*_, 0.5; H_2_/CO_*x*_, 3; TOS, 16 h.

As previously mentioned, one of the objectives of this work is to assess the perspectives of cofeeding CO_2_ and syngas. With this purpose, the conversions of CO_2_ and CO_*x*_ (CO + CO_2_) are compared in Figure 6. The results correspond to the values after 16 h time on stream, that is, for the pseudosteady state of the catalyst. Comparing these results with olefin yields under the same conditions permits distinguishing between the conversion of CO_2_ into olefins or into CO. This trend is difficult to predict, as raising temperature favors CO formation through the rWGS reaction and also the conversion of methanol/DME into olefins. The results in Figure 6 show the increase in CO_2_ when raising reaction temperature within the 375–425 °C range, whereas the maximum CO_*x*_ conversion takes place at 400 °C as olefin yield is maximized (Figure 3). Consequently, upon increasing the temperature from 375 to 400 °C, olefin formation is favored to a greater extent than that of CO, whereas the trend reverts at 425 °C. This result is in accordance with the literature on methanol synthesis (with CuO-ZnO-Al_2_O_3_ catalysts essentially), which establishes the greater reactivity of CO_2_ with respect to CO at low conversion conditions (low concentration of H_2_O), situations in which the active sites are not blocked by the product H_2_O, whereas the results invert for higher conversions.^32^ This is also in accordance with that reported by Tsoukalou et al. for In_2_O_3_ catalysts.^47^ Indeed, the higher reactivity of CO_2_ at these conditions is responsible for the lower olefin/paraffin ratio (O/P) in this work compared with similar studies in the literature, as CO_2_+CO mixtures are used as carbon source in our case, unlike the pure CO_2_ feedstocks used in the literature.

These results show that CO_2_+CO mixtures in the feedstock do not hamper CO_2_ conversion, meaning that the approach presented in the work, of considering H_2_+CO+CO_2_ feedstocks feasible for the process, is viable, as similar values were obtained by other authors within the 360–400 °C range with H_2_+CO_2_ feedstocks over In_2_O_3_/SAPO-34^65^ and In_2_O_3_–ZrO_2_/SAPO-34^48^ catalysts.

#### 3.1.3 Deactivation of the Catalyst by Coke

**Figure 7:**
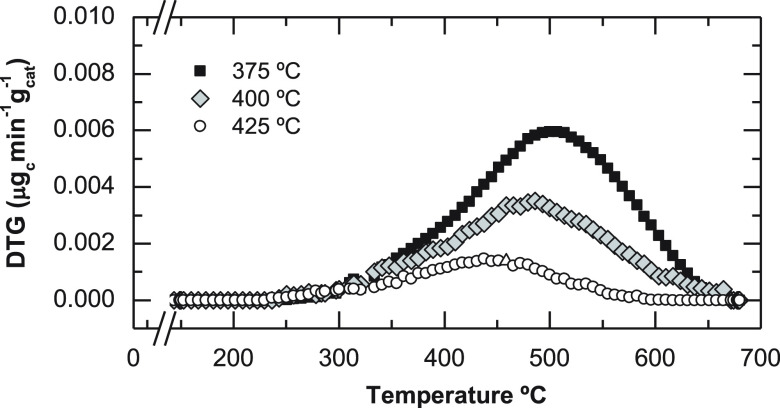
TPO profiles of the coke deposited on SAPO-34 at different reaction temperatures. Reaction conditions: 30 bar; 5 g_cat_ h mol_C_^–1^; CO_2_/CO_*x*_, 0.5; H_2_/CO_*x*_, 3; TOS, 24 h.

As previously stated, catalyst deactivation is attributable to the fast deposition of coke. Given that the bifunctional InZr/S34 catalyst was prepared by a physical mixture of different sized particles of each function, separately analyzing the coke content deposited in In_2_O_3_–ZrO_2_ and SAPO-34 is feasible. The temperature programmed oxidation analyses reveal that the coke content deposited on the In_2_O_3_–ZrO_2_ function is negligible compared to that deposited over the SAPO-34 (Figure S2). Therefore, in Figure 7, the TPO profiles for the SAPO-34 acid catalysts used in the experiments described in Figures 2 and 3 are presented. The total coke content in the acid catalyst (calculated as the area under the TPO profile, Figure 7) is very high at 375 °C (15.4 wt %), whereas it diminishes remarkably with increasing reaction temperature (9.0 wt % at 400 °C and 3.7 wt % at 425 °C). The coke content reduction when raising reaction temperature is consistent with the lower deactivation observed at higher reaction temperature (Figures 2 and 3). In the TPO profiles, a broad range of combustion temperature is observed, revealing a heterogeneous composition of coke.^72,73^ The maximum combustion rate temperature (peak in the 450–500 °C range) is consistent with coke deposited in the porous structure of SAPO-34.^74^ The decrease in the temperature required for the maximum combustion rate upon increasing the reaction temperature is in accordance with the hypothesis that the coke is composed of less condensed species, because of the greater extent of the hydrogenation of coke precursors. These hydrogenation reactions will presumably be activated by the metallic sites of the catalyst.

It is well established in the literature that the fast coke formation over SAPO-34 in the processes for methanol/DME conversion to hydrocarbons, whose mechanism occurs via condensation to polyaromatic structures of the intermediates (polymethylbenzenes and olefins), is due to reactions catalyzed by strong acid sites.^75^ The microporous structure of SAPO-34, with cages in the intersections of the crystalline channels, favors the confinement of these polyaromatics, blocking access to the acid sites. The faster decay of light paraffin yield over that of olefins at the beginning of the reaction, at short time on stream values and 375 and 400 °C (Figure 3a, b, respectively), can be related to a minimum coke deposition in the In_2_O_3_–ZrO_2_ function. This incipient coke formation has been explained by the presence of formaldehyde and methoxy ions as intermediates for bifunctional catalysts prepared with CuO as metallic function and used in the direct synthesis of DME from CO+CO_2_ mixtures.^76^ Likewise, analogous coke deposition is observed in Cu^29^ and Ni–In^76^ catalysts used in methanol synthesis from CO_2_, although this deposition is attenuated by the presence of H_2_O in the medium, which is greater using CO_2_ as a reactant than using CO.^77^ The hydrocarbons (at low concentration) resulting from side reactions in methanol/DME synthesis also act as precursors of the coke deposited on the acid function.^78^

As previously mentioned, acquiring a pseudostable state of constant activity is important for the viability of the catalyst (Figures 2 and 3). This result is explained by the hydrogenation of the intermediate precursors of coke. These equilibrium of coke deposition on SAPO-34 in a H_2_ atmosphere at high pressure has been proven for the MTO process^79^ and for the direct synthesis of hydrocarbons from syngas.^53^ The content of H_2_O in the reaction medium will also contribute to the attenuation of coke formation, by competing for its adsorption in the acid sites of the catalyst with the precursors of coke.^35^ This effect justifies cofeeding H_2_O with methanol in the MTO process.^52^

### 3.2 Pressure

**Figure 8:**
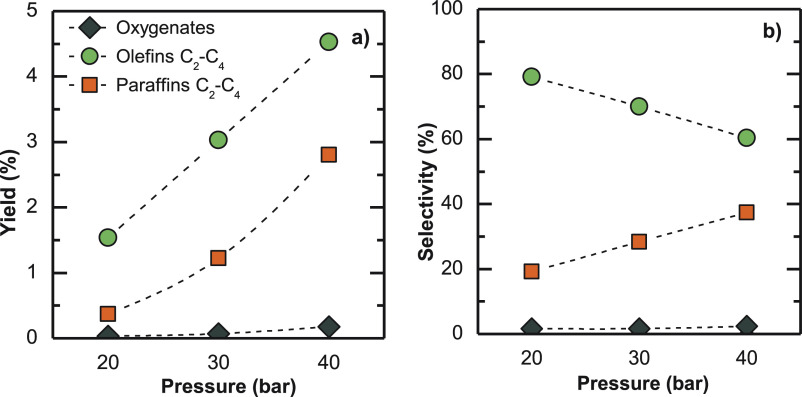
Pressure effect on product (a) yields and (b) selectivities. Reaction conditions: 400 °C; 5 g_cat_ h mol_C_^–1^; CO_2_/CO_*x*_, 0.5; H_2_/CO_*x*_, 3; TOS, 16 h.

The results of hydrocarbon yields and selectivities gathered in Figure 8 correspond to three different operating pressures. As expected, pressure favors methanol/DME synthesis reactions, resulting in an olefin yield boost. Analogously, hydrogenation reactions are also favored. Consequently, paraffin yield is also promoted when the reaction pressure is praised (Figure 8a). As an overall result, olefin selectivity decreases and that of paraffins increases (Figure 8b). It should be noted that oxygenate yield in the product stream is insignificant (0.17%) at 40 bar, as the dehydration of methanol/DME is disfavored with increasing reaction pressure. The increase in propylene selectivity (Figure S3) is consistent with the known effect of pressure (according to the dual cycle mechanism hypothesis) on favoring the advance of the alkene cycle (with oligomerization/cracking reactions) with respect to that of aromatics, in which ethylene is the main product of methylation/dealkylation reactions.^69^ The cracking of butenes to ethylene will also be disfavored with increase pressure, which explains the gain in the selectivity of the former.

**Figure 9:**
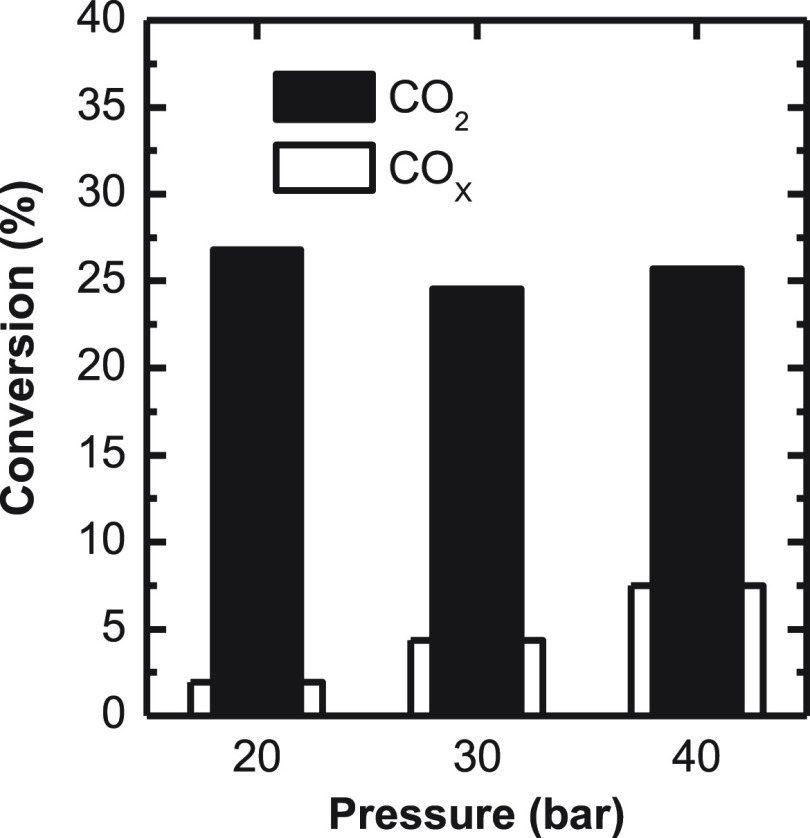
Pressure effect on CO_*x*_ and CO_2_ conversion. Reaction conditions: 400 °C; 5 g_cat_ h mol_C_^–1^; CO_2_/CO_*x*_, 0.5; H_2_/CO_*x*_; 3; TOS, 16 h.

In Figure 9, it is observed that pressure increase has a low impact on the conversion of CO_2_, whereas it notably promotes the conversion of CO_X_, tripling its value from 1.9% at 20 bar to 7.5% at 40 bar. This effect can be attributed to the competition of CO_2_ and CO for the adsorption in the active sites. The results indicate that in this competition the adsorption of CO is selectively favored, increasing the formation rate of formyl and carboxyl ions, which are methanol formation intermediates.^77^

### 3.3 Space Time

**Figure 10:**
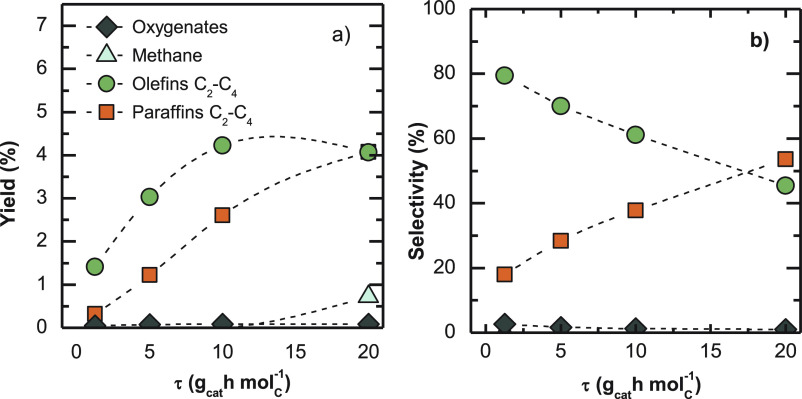
Space time effect on product (a) yields and (b) selectivities. Reaction conditions: 400 °C; 30 bar; CO_2_/CO_*x*_, 0.5; H_2_/CO_*x*_, 3; TOS, 16 h.

As olefins are intermediate products in the conversion of methanol/DME into hydrocarbons (as they tend to hydrogenate to paraffins in such a high hydrogen partial pressure environment), ascertaining an optimal space time for their production is critical. As shown in Figure 10a, higher space time values lead to higher yields of olefins and paraffins, even if following different trends. Olefin yield tends to an asymptote around 4.5%, whereas paraffins show a more constant increment in the studied range of space time. Consequently, as can be observed in Figure 10b, paraffin selectivity grows as space time values increase, to the detriment of olefin selectivity. It is also observed that for high space time values, methane formation is outstanding (Figure 10a). Among the different causes of methane formation (cracking of olefins, decomposition of methanol/DME), cracking seems more likely, because oxygenate concentration is negligible for high space time values, and it is well established that the increase in space time favors olefin cracking.^70^ This methane formation by butene cracking is also consistent with the increase in propylene selectivity with increasing space time.

As to the effect on olefin distribution (Figure S4), the ethylene/propylene ratio decays slightly upon increasing space time, which favors the alkene cycle.^69^ The decrease in butene concentration is noteworthy for high space time (20 g_cat_ h mol_C_^–1^), probably due to their cracking, which is consistent with the aforementioned significant presence of methane in these conditions. Anyhow, the results obtained at low space time values (below 10 g_cat_ h mol_C_^–1^) show a higher O/P ratio for a certain value of olefin yield compared with other works in the literature. Numpilai et al.^65^ obtained an O/P ratio close to 1 for olefin yield values around 3% with an In_2_O_3_/SAPO-34 catalyst at 400 °C, 25 bar, and 16.3 g_cat_ h mol_C_^–1^ (equivalent to GHSV of 6000 mL h^–1^ g_cat_^–1^, with H_2_+CO_2_ feedstocks). In the present work, such olefin yield may be achieved at 5 g_cat_ h mol_C_^–1^, reaching an O/P ratio of 2.5.

**Figure 11:**
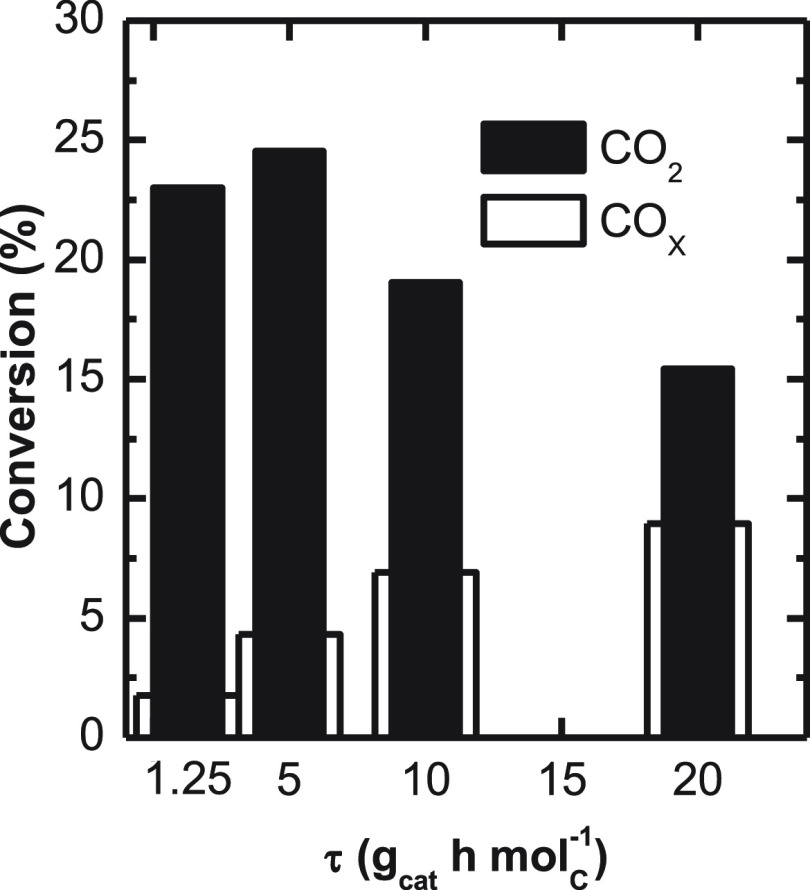
Space time effect on CO_*x*_ and CO_2_ conversion. Reaction conditions: 400 °C; 30 bar; CO_2_/CO_*x*_, 0.5; H_2_/CO_*x*_, 3; TOS, 16 h.

In Figure 11, the conversions of CO_2_ and CO_*x*_ are compared for various space time values. It is observed that the conversion of CO_*x*_ increases continuously with increasing space time. Taking into account that the conversion of CO_2_ passes through a maximum at a low value of space time (under 5 g_cat_ h mol_c_^–1^), this result seems to indicate that for low space time values the main source of carbon of the olefins is CO_2_, being the corresponding mechanism favored with respect to the hydrogenation of CO. Thus, for low space time (5 g_cat_ h mol_c_^–1^) the results in Figures 6 and 9, corresponding to different temperatures and pressures, respectively, have also revealed the greater reactivity of CO_2_ under these conditions of incipient hydrogenation of CO and CO_2_. This apparent discrepancy with respect to the different reactivity of CO and CO_2_ has been the subject of controversy in the literature on methanol synthesis. For CuO-ZnO-Al_2_O_3_ catalysts, Nielsen et al.^32^ give an explanation for this discrepancy, relating CO_2_ reactivity with methanol content (and consequently with H_2_O) in the reaction medium, that is, lower methanol concentration resulting in higher CO_2_ reactivity. However, according to these authors, this occurs through the rWGS reaction, avoiding the unfavorable effect of H_2_O on attenuating the activity of the Cu sites. The results of the present work, obtained by feeding CO_2_ together with syngas, are consistent with this explanation and with the results for CO_2_ hydrogenation on In_2_O_3_ catalysts reported by different authors^47,59^ who have obtained a lower apparent activation energy for CO_2_ hydrogenation than for the rWGS reaction at high pressure, low space time values, and mild reaction temperature conditions.

### 3.4 H_2_/CO_*x*_ Ratio in the Feed

**Figure 12:**
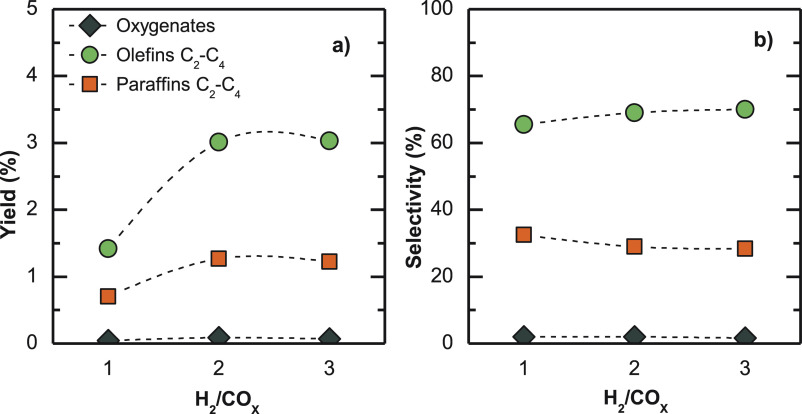
H_2_/CO_*x*_ molar ratio in the feed effect on product (a) yields and (b) selectivities. Reaction conditions: 400 °C; 30 bar; 5 g_cat_ h mol_C_^–1^; CO_2_/CO_*x*_, 0.5; TOS, 16 h.

The results obtained for different H_2_/CO_*x*_ molar ratios in the feed (Figure 12) on hydrocarbon production show the need for using a ratio of 2 to activate CO hydrogenation reactions. This ratio is stoichiometric for CO_2_ hydrogenation, and further increasing this molar ratio does not lead to any improvement on olefin yields (Figure 12a). Consequently, a H_2_/CO_X_ molar ratio of 2 is set as optimal for attending to the economic criteria of H_2_ cost and to the possibility of obtaining the required H_2_+CO+CO_2_ feedstock mixture from a wider variety of processes, including biomass gasification or reforming of its derivatives (methanol, ethanol, bio-oil).

In addition, it is observed in Figure 12b that increasing the H_2_/CO_*x*_ ratio between 1 and 3 does not have effects on olefin selectively, meaning that hydrogenation reactions are not favored. In fact, O/P ratio does even increase to some extent. This result indicates that under these conditions the overall reaction is limited by the advance of methanol/DME synthesis and the space time is low enough to limit olefin hydrogenation. Furthermore, according to Figure S5, increasing methanol/DME concentration by raising the H_2_/CO_*x*_ ratio results in a change in olefin distribution. Within the studied range, increasing the H_2_/CO_*x*_ ratio the concentration of butenes in the product stream decreases and the ethylene/propylene ratio also diminishes in the H_2_/CO_*x*_ 1 to 2 interval. This result indicates that H_2_-rich feedstocks favor the change in the cycle of alkenes in the formation of olefins, with respect to the cycle of aromatics.^69^

**Figure 13:**
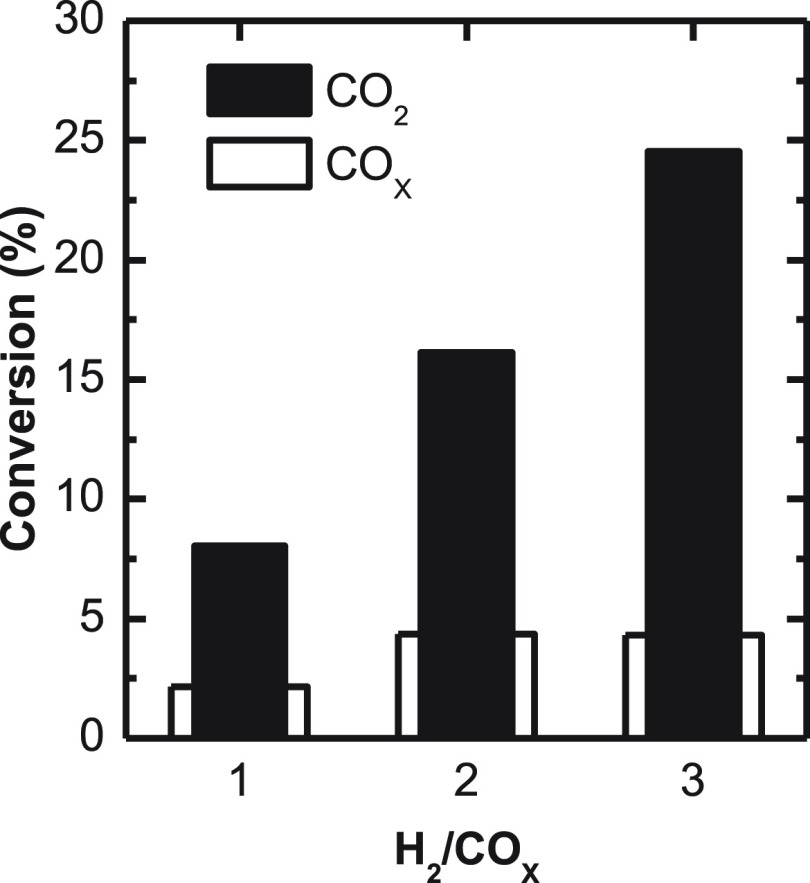
H_2_/CO_*x*_ molar ratio in the feed effect on CO_*x*_ and CO_2_ conversion. Operating conditions: 400 °C, 30 bar, 5 g_cat_ h mol_C_^–1^; CO_2_/CO_*x*_, 0.5, TOS = 16 h.

In Figure 13, the evolution of CO_2_ and CO_*x*_ conversions with the H_2_/CO_*x*_ ratio are compared. The continuous gain of CO_2_ conversion observed when increasing this ratio can be explained by methanol synthesis from CO_2_ along with CO_2_ conversion into CO through the rWGS reaction. The increase in CO_*x*_ conversion with increasing the H_2_/CO_*x*_ ratio from 1 to 2 supports that both effects take place. However, by increasing the ratio from 2 to 3, the conversion of CO_*x*_ remains constant, indicating that further increasing the H_2_/CO_*x*_ ratio only affects the rWGS reaction, favoring its advance.

## 4 Conclusions

The direct synthesis of light olefins from CO_2_ and syngas mixture hydrogenation is an attractive alternative to the two-stage process, because it can be carried out under low H_2_ pressure conditions and a moderate H_2_/CO_*x*_ ratio, facilitating the valorization of syngas obtained from biomass or wastes and H_2_ generated with sustainable energy sources. The In_2_O_3_–Zr_2_O_3_/SAPO-34 catalyst is active for the conversion of CO_2_ and selective to olefins and is remarkably stable at 400 °C because of the fact that after an initial deactivation period, the formation of coke is prevented by the hydrogenation of the precursors for its formation, so that the catalyst acquires a constant activity. Space time has an important effect on the relative reactivity of CO_2_ and CO. Thus, the greatest CO_2_ reactivity is achieved at low space time values, whereas CO_*x*_ conversion follows a growing trend with increasing space time, since the conversion of CO is favored.

The extent of the reaction and thus olefin yield are limited by the thermodynamics of the methanol/DME synthesis step and conditioned by the influence of reaction conditions on the rWGS reaction and on the extent of the dual cycle of methanol/DME conversion into olefins. In this stage, 400 °C and low values of pressure (20–30 bar) and space time (5–10 g_cat_ h mol_C_^–1^) are the suitable conditions for valorizing the CO_2_ fed together with syngas, resulting in light olefin yield over 4% and high selectivity (70–80%), with light paraffins as byproducts. Olefin distribution is propylene > ethylene> butenes. For suitable conditions (400 °C, 30 bar, 5–10 g_cat_ h mol_C_^–1^, CO_2_/CO_*x*_ = 0.5, and H_2_/CO_*x*_ = 3), a propylene/ethylene/butene ratio (%) of 35/53/12 is obtained.

The results of this work, studying separately the effect of the reaction variables on the conversion of CO_2_ and of CO_*x*_ (CO_2_ + CO), show that the syngas cofeeding does not have an unfavorable effect on the main objective of CO_2_ conversion and thus that the cofeeding strategy is viable.

## Supporting Information Available

Additional results, influence of temperature on product selectivity evolution with TOS; TPO; influence of pressure, space time, and fed H_2_/CO_*x*_ ratio on olefin distribution (PDF)
