# Supplementary material for: Conditions for the Joint Conversion of CO2 and Syngas in the Direct Synthesis of Light Olefins Using In2O3–ZrO2/SAPO-34 Catalyst
Source: Ind Eng Chem Res. 2021 Nov 9;61(29):10365–76. doi: 10.1021/acs.iecr.1c03556 (PMC9335533; doi:10.1021/acs.iecr.1c03556)
Supplement: Supplementary file 1 — ie1c03556_si_001.pdf [file ie1c03556_si_001.pdf]

Supporting Information for:

Conditions for the joint conversion of CO_2 and syngas in the direct synthesis of light olefins using In_2O_3-ZrO_2/SAPO-34 catalyst

Ander Portillo, Ainara Ateka*, Javier Ereña, Andres T. Aguayo, Javier Bilbao

Department of Chemical Engineering, University of the Basque Country UPV/EHU, P.O. Box 644, 48080 Bilbao, Spain

*Corresponding author. *Tel.:* 34-94-6015341. *E-mail address*: ainara.ateka@ehu.eus

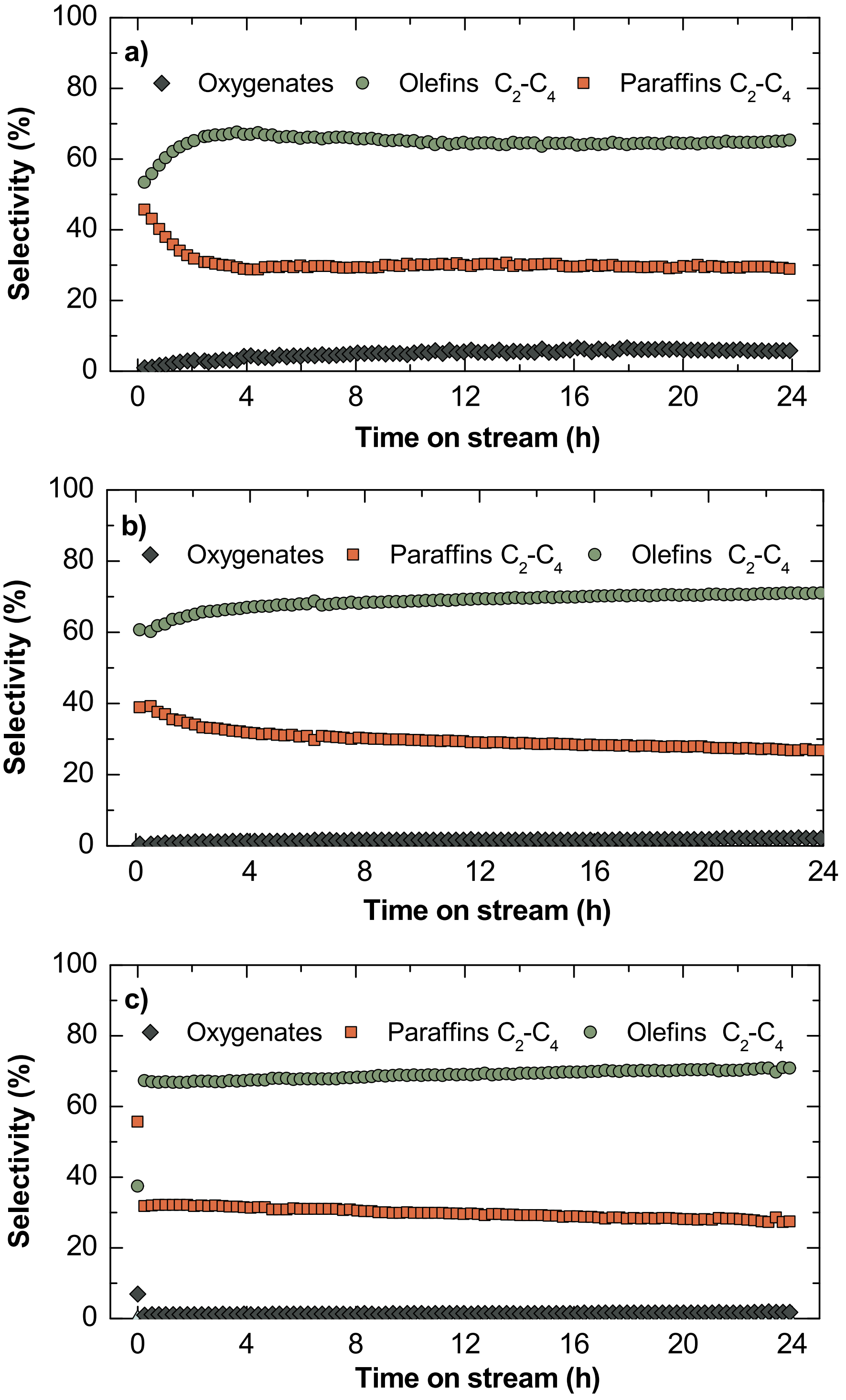

Fig. S1. Evolution of the selectivity of products with time on stream at 375 ºC (a), 400 ºC (b) and 425 ºC (c). Reaction conditions: 30 bar; 5 g_{cat} h mol_C^{-1}; CO_2/COx, 0.5; H_2/CO_x, 3; TOS, 24 h.

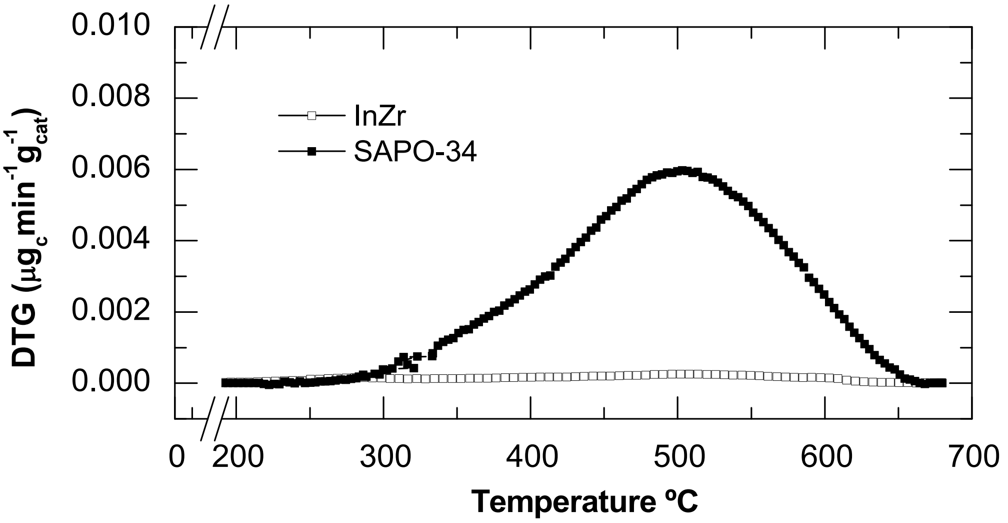

Figure S2. TPO profile of the coke deposited on the In_2O_3-ZrO_2 metallic and SAPO-34 acid functions of the bifunctional catalyst. Reaction conditions: 400 ºC; 30 bar; 5 g_{cat} h mol_C^{-1}; CO_2/COx, 0.5; H_2/CO_x, 3; TOS, 24 h.

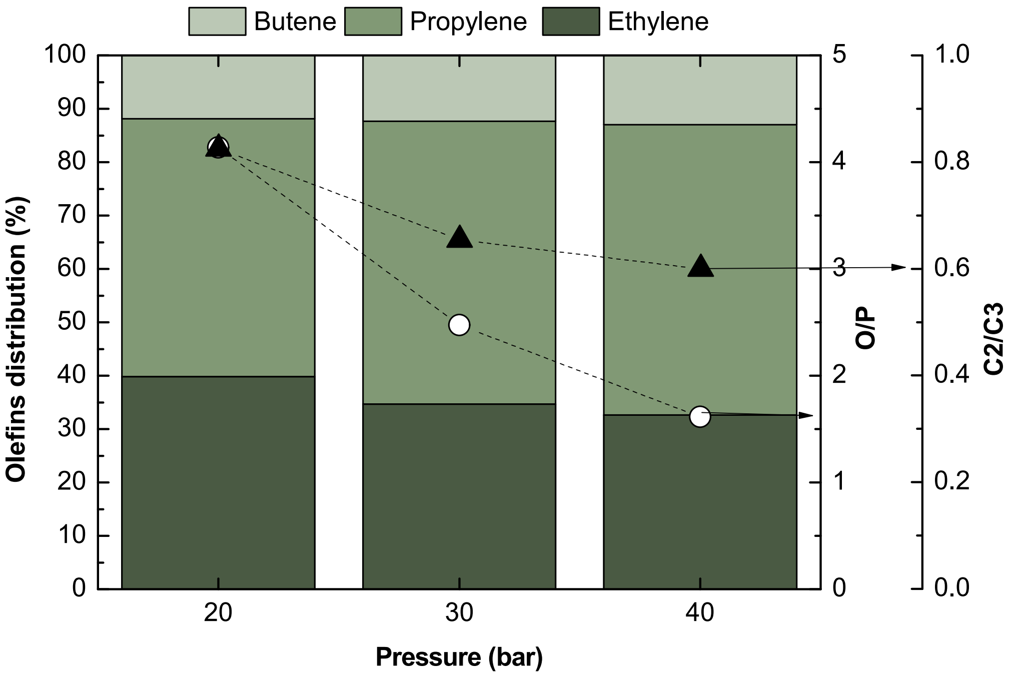

Fig. S3. Olefins distribution for different reactions pressures. Operating conditions: 400 ºC; 5 g_{cat} h mol_C^{-1}; CO_2/COx, 0.5; H_2/CO_x, 3; TOS, 16 h.

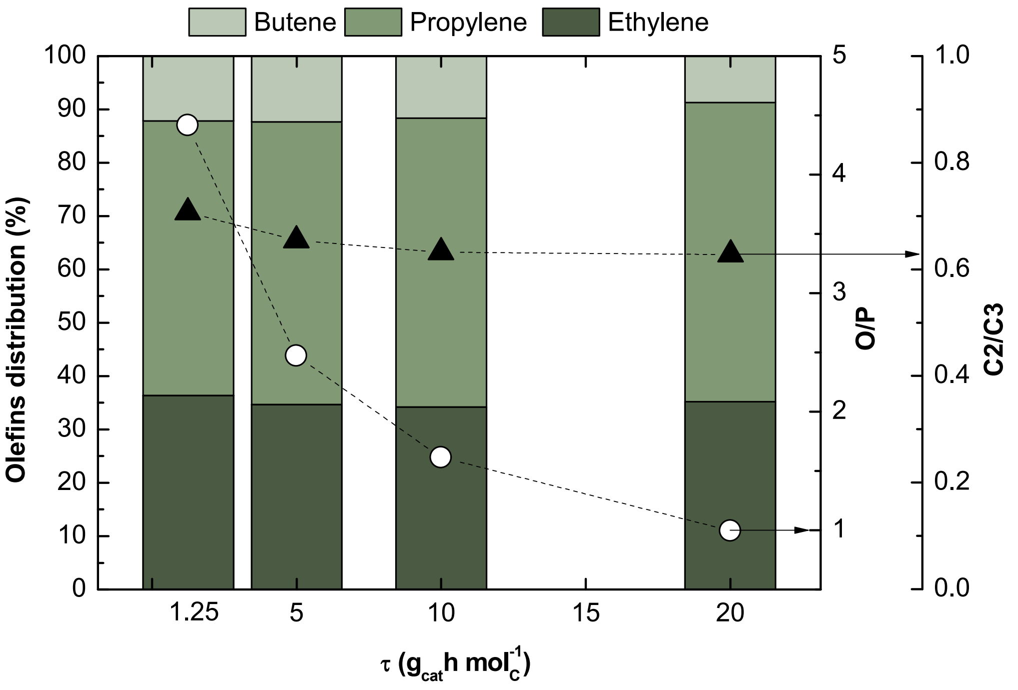

Fig. S4. Effect of space time on olefins distribution Reaction conditions: 400 ºC; 30 bar; CO_2/COx, 0.5; H_2/CO_x, 3; TOS, 16 h.

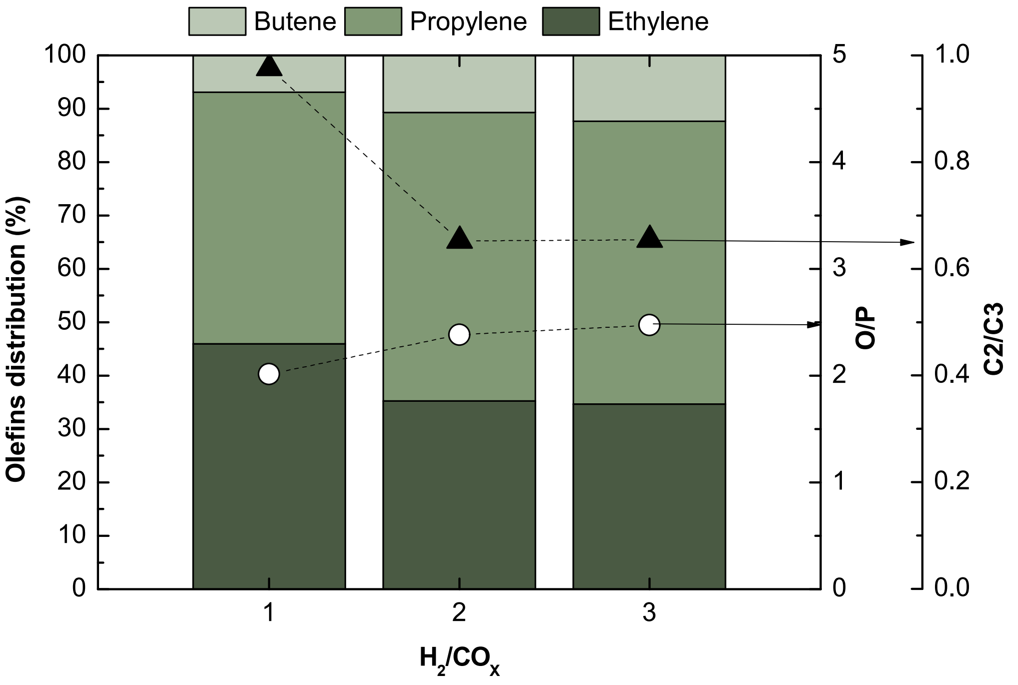

Fig. S5. H_2/CO_x molar ratio effect on olefins distribution. Reaction conditions: 400 ºC; 30 bar; 5 g_{cat} h mol_C^{-1}; CO_2/COx, 0.5; TOS, 16 h.
